# Infection with a Brazilian isolate of Zika virus generates RIG‐I stimulatory RNA and the viral NS5 protein blocks type I IFN induction and signaling

**DOI:** 10.1002/eji.201847483

**Published:** 2018-04-06

**Authors:** Jonny Hertzog, Antonio Gregorio Dias Junior, Rachel E. Rigby, Claire L. Donald, Alice Mayer, Erdinc Sezgin, Chaojun Song, Boquan Jin, Philip Hublitz, Christian Eggeling, Alain Kohl, Jan Rehwinkel

**Affiliations:** ^1^ Medical Research Council Human Immunology Unit Medical Research Council Weatherall Institute of Molecular Medicine Radcliffe Department of Medicine University of Oxford Oxford UK; ^2^ MRC‐University of Glasgow Centre for Virus Research Glasgow Scotland UK; ^3^ Genome Engineering Facility Medical Research Council Weatherall Institute of Molecular Medicine Radcliffe Department of Medicine University of Oxford Oxford UK; ^4^ Department of Immunology The Fourth Military Medical University Xi'an PR China

**Keywords:** Interferon, MDA5, RIG‐I, STAT, Zika virus

## Abstract

Zika virus (ZIKV) is a major public health concern in the Americas. We report that ZIKV infection and RNA extracted from ZIKV infected cells potently activated the induction of type I interferons (IFNs). This effect was fully dependent on the mitochondrial antiviral signaling protein (MAVS), implicating RIG‐I‐like receptors (RLRs) as upstream sensors of viral RNA. Indeed, RIG‐I and the related RNA sensor MDA5 contributed to type I IFN induction in response to RNA from infected cells. We found that ZIKV NS5 from a recent Brazilian isolate blocked type I IFN induction downstream of RLRs and also inhibited type I IFN receptor (IFNAR) signaling. We defined the ZIKV NS5 nuclear localization signal and report that NS5 nuclear localization was not required for inhibition of signaling downstream of IFNAR. Mechanistically, NS5 blocked IFNAR signaling by both leading to reduced levels of STAT2 and by blocking phosphorylation of STAT1, two transcription factors activated by type I IFNs. Taken together, our observations suggest that ZIKV infection induces a type I IFN response *via* RLRs and that ZIKV interferes with this response by blocking signaling downstream of RLRs and IFNAR.

## Introduction

Viruses from the *Flaviviridae* family are enveloped and contain a positive sense, single stranded RNA genome. This virus family includes many pathogens important to human health such as hepatitis C virus and mosquito‐borne dengue virus (DENV), West Nile virus and Zika virus (ZIKV). ZIKV was initially described in 1947 after isolation from monkeys living in the Zika forest in Uganda 1, 2 but has only received notable attention since the recent epidemic in Brazil and other parts of the Americas. ZIKV can be categorized into viruses of the Asian and African lineage. The 2015/2016 epidemic strain belongs to the Asian lineage and shares a common ancestor with viruses causing outbreaks in Polynesia in 2013/2014 3, 4. Infection is often asymptomatic or is characterized by a self‐limiting acute febrile illness, including mild fever, maculopapular rash, arthralgia and conjunctivitis 5, 6. In adults, ZIKV infection has also been suggested to trigger Guillain‐Barré syndrome, a rapid‐onset muscle weakness caused by an autoimmune response 7. ZIKV is most commonly transmitted by *Aedes* mosquitoes; however, other routes of transmission include sexual and maternal‐fetal during pregnancy 8, 9, 10. In the latter case, *in utero* ZIKV infection may cause developmental defects resulting in microcephaly 11. Indeed, the recent epidemic coincided and overlapped geographically with an increase in microcephaly cases in newborns 3. Moreover, ZIKV infects neural progenitor cells and vertical transmission as well as fetal microcephaly have been documented in mouse models [reviewed in: 7].

Type I interferons (IFNs, including IFN‐α and IFN‐β) are cytokines which coordinate many aspects of the mammalian immune response to infectious microorganisms 12. During viral infections, type I IFNs are often crucial to successful immunity. In the course of an infection, their expression is induced at the transcriptional level in different types of cells. This occurs downstream of pathogen sensing by innate immune receptors 13, 14. Sensors of virus presence often detect nucleic acids as molecular signatures of infection; for example, viral RNA or DNA are potent triggers for type I IFN induction 13, 14. These sensors include toll‐like receptors, which survey the endosomal compartment, as well as cytosolic DNA receptors and RIG‐I‐like receptors (RLRs) that are localized in the cytosol of cells 13, 14. RLRs are helicase proteins and include RIG‐I and MDA5. RIG‐I recognizes viral RNAs that have uncapped 5’‐ends marked by tri‐ or diphosphate groups 15, 16. Upon binding to viral or unusual RNAs, RIG‐I and MDA5 engage the adaptor protein MAVS. Signaling downstream of MAVS activates transcription factors including IRF3 and NF‐κB, which then drive transcription of the genes encoding type I IFNs and other antiviral genes 13, 14. Once secreted, type I IFNs bind to the dimeric type I IFN receptor (IFNAR) on the same or other cells 12. This results in activation of JAK1 and TYK2 kinases, which in turn phosphorylate and thereby activate STAT1 and STAT2. These transcription factors then form a complex with IRF9 and induce the expression of hundreds of interferon‐stimulated genes (ISGs). The proteins encoded by ISGs have a variety of direct and indirect antiviral effects 17.

ZIKV infection in cultured human cells and mice is controlled by type I IFNs. For example, treatment of human skin fibroblasts or A549 cells with IFN‐α or IFN‐β prior to infection diminishes ZIKV replication 18, 19. In vivo, ZIKV infection in mice is restricted by type I IFNs. Virus replication, transmission and pathology are exacerbated in animals lacking IFNAR, STAT2, MAVS or IRF transcription factors and after treatment with an IFNAR blocking antibody 20, 21, 22, 23, 24, 25, 26. Interestingly, in human monocyte‐derived dendritic cells and skin fibroblasts, the addition of type I IFN after or concomitantly with infection only modestly reduces ZIKV replication 18, 27, suggesting viral interference with the effects of type I IFNs. Indeed, recent studies have reported that ZIKV NS5 proteins from African and French Polynesian isolates antagonize IFNAR signaling by targeting STAT2 for degradation 19, 28. However, NS5 and other ZIKV proteins from virus strains associated with the latest outbreak in Brazil have not yet been analyzed in this context, which is important given that Zika virus isolates differ significantly in in vivo and in vitro infection settings 29, 30. Additionally, ZIKV infection has been suggested to prevent STAT1 and STAT2 phosphorylation 27.

We addressed the open question of how ZIKV is detected by infected cells to induce type I IFNs. We report that ZIKV infected cells accumulated RNA molecules that potently activated RIG‐I in a 5’‐phosphate‐dependent manner. MDA5‐stimulatory RNAs were also generated during infection, suggesting that ZIKV infection is sensed by RLRs. Based on sequence information from a Brazilian ZIKV isolate, we individually cloned all viral proteins into a mammalian expression vector. We demonstrate that NS5 from a Brazilian 2015/2016 epidemic strain of ZIKV blocks type I IFN induction mediated by RLRs and IFNAR signaling. The latter function of NS5 involved depleting STAT2 levels and preventing phosphorylation of STAT1. Nuclear localization was not required for this function of NS5. We propose a model in which ZIKV induces a type I IFN response *via* cytosolic RNA sensors of the RLR family and antagonizes this response by blocking signaling downstream of RLRs and IFNAR.

## Results

### ZIKV infection induces type I IFN and generates RLR‐stimulatory RNA

**Figure 1:**
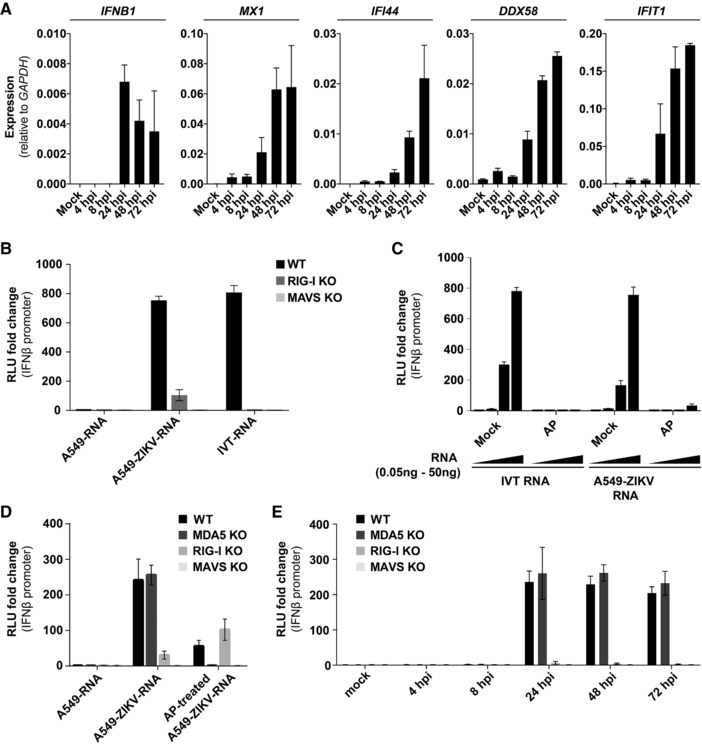
ZIKV infection generates RIG‐I‐ and MDA5‐stimulatory RNAs. (A) A549 cells were infected with ZIKV (MOI of 5) and total RNA was extracted at the indicated time points. Expression levels of the indicated mRNAs were determined by RT‐qPCR with samples assayed in technical duplicates. Data were analyzed by the comparative C_t_ method. (B) A549 cells were infected with ZIKV (MOI of 5) and total RNA was extracted after 20 h (A549‐ZIKV‐RNA). 100 ng A549‐ZIKV‐RNA or RNA from uninfected cells (A549‐RNA) was then transfected into the indicated p125HEK cells (see Supporting Information Fig. 1). After 16 h, luciferase activity was determined. Data are shown as fold change compared to cells treated with transfection reagent only. IVT‐RNA, Neo^1‐99^ in vitro transcribed RNA (RIG‐I agonist); RLU, relative light unit. (C) IVT‐RNA and A549‐ZIKV‐RNA were treated with alkaline phosphatase (AP). Control samples were processed in parallel omitting AP (mock). These RNAs were then analyzed as in (B) using wild‐type p125HEK cells and four doses of RNA (0.05, 0.5, 5, and 50 ng). (D) The experiment shown in (B) was repeated using the indicated p125HEK cell lines that were pre‐treated with 3 U/ml IFN‐A/D for 16 h and were then transfected with 50 ng of total RNA from uninfected cells (A549‐RNA) or with A549‐ZIKV‐RNA that was or was not treated with AP. (E) The experiment shown in (B) was repeated using A549‐ZIKV‐RNA isolated at the indicated time points after infection. Panel (A) shows pooled data of two independent experiments (average and SEM (*n* = 4)). Panel (B) is representative of three independent experiments (average and SD (*n* = 3)). Panels (C) and (D) are representative of two independent experiments (average and SD (*n* = 3)). Panel (E) shows pooled data of two independent experiments (average and SEM (*n* = 6)).

Infection of cells with RNA viruses often results in RIG‐I‐ and/or MDA5‐dependent type I IFN production, and infected cells accumulate RLR‐stimulatory RNA molecules. Experimentally, this can be tested by the extraction of RNA from infected cells followed by transfection of reporter cells. For example, IAV infected cells accumulate RIG‐I‐stimulatory RNAs and encephalomyocarditis virus (EMCV) infection results in the generation of extractable RNAs that activate MDA5 31, 32. This assay therefore faithfully predicts which RLRs are involved in detecting the virus in the infected cell 33. To test this in the context of ZIKV, we infected A549 cells with a ZIKV strain from Brazil (PE243, 34) at a multiplicity of infection (MOI) of 5 and extracted total RNA at different time points post infection. We first tested whether a type I IFN response was induced in this setting. Indeed, using RT‐qPCR, we found that *IFNβ* mRNA and several transcripts encoded by ISGs were upregulated in ZIKV‐infected A549 cells (Fig. 1A). Next, we transfected RNA from A549 cells infected with ZIKV for 20 hours (A549‐ZIKV‐RNA) into HEK293 cells stably expressing firefly luciferase (F‐Luc) under control of the *IFNβ* promoter (Supporting Information Fig. S1A‐C). A549‐ZIKV‐RNA induced expression of the *IFNβ* reporter and the magnitude of the response was similar to that triggered by Neo^1‐99^ in vitro transcribed (IVT) RNA, a 5’‐triphosphate containing RIG‐I agonist 31 (Fig. 1B). RNA from uninfected A549 cells did not induce the *IFNβ* promoter in these settings (Fig. 1B). Next, we analyzed the response in RIG‐I or MAVS knockout reporter cells (Supporting Information Fig. S1D‐O). As expected, the response to IVT‐RNA was fully RIG‐I‐ and MAVS‐dependent (Fig. 1B). Induction of the *IFNβ* reporter after A549‐ZIKV‐RNA transfection was strongly reduced in RIG‐I‐deficient cells and was completely absent in MAVS knockout cells (Fig. 1B). To corroborate this finding, we treated A549‐ZIKV‐RNA and IVT‐RNA as a control with alkaline phosphatase (AP), which removes the 5’‐phosphates required for RIG‐I activation 31. Indeed, AP treatment abolished IFN induction in response to IVT‐RNA, and largely prevented the response to A549‐ZIKV‐RNA (Fig. 1C). These results show that A549‐ZIKV‐RNA contains RIG‐I‐stimulatory RNA molecules. To test whether the remaining response to A549‐ZIKV‐RNA observed in RIG‐I‐deficient cells or after AP treatment could be attributable to MDA5, we included MDA5 knockout cells in the analysis (Supporting Information Fig. 1). Indeed, the response to AP‐treated A549‐ZIKV‐RNA was MDA5‐dependent (Fig. 1D). We also analyzed the kinetics of RLR‐stimulatory RNA accumulation in ZIKV‐infected cells and found this had occurred between 8 and 24 h post‐infection (Fig. 1E), correlating with the time course of *IFNβ* and ISG mRNA induction in infected cells (Fig. 1A). This therefore suggests that viral replication might be required to activate RLRs. Taken together, these observations show that ZIKV infection results in the accumulation of RIG‐I‐ and MDA5‐stimulatory RNAs.

**Figure 2:**
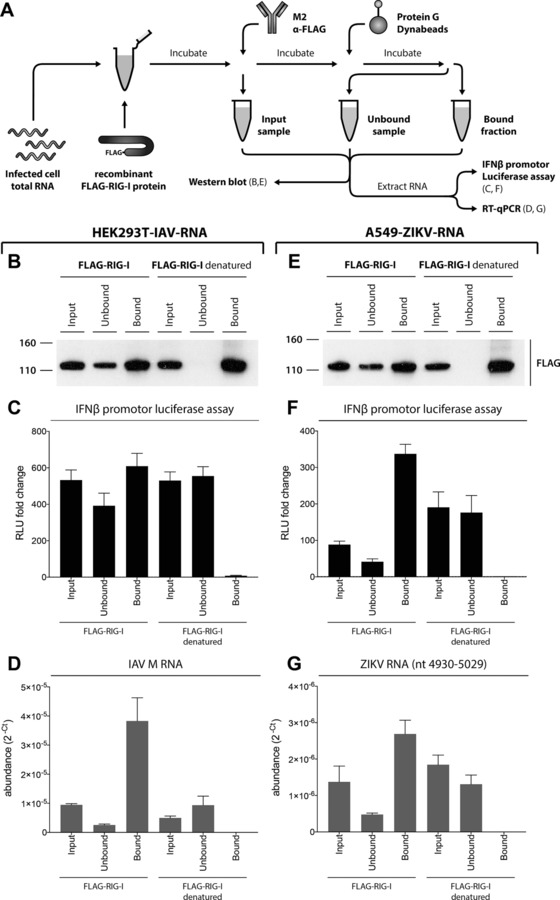
RIG‐I binds ZIKA RNA. (A) Schematic of the experimental setup. Please see results for detail. HEK293T‐IAV‐RNA (panels B–D) and A549‐ZIKV‐RNA (E–G) were used. (B, E) Input, unbound and bound samples were analyzed by Western blot using α‐FLAG antibody. (C, F) RNAs extracted from input, unbound and bound samples were transfected into p125HEK cells (see Supporting Information Fig. 1). After 16 h, luciferase activity was determined and set to 1 for control samples treated with transfection reagent alone. RLU, relative light unit. (D, G) RNA samples from (C) and (F) were analyzed in technical duplicate by RT q‐PCR using the indicated probes. Relative viral transcript abundance was analyzed by converting C_t_ data to 2^−Ct^ values. Panels (B) and (E) are representative of three independent experiments. Panels (C), (D), (F) and (G) show pooled data from three independent experiments (average and SEM (*n* = 6)).

We next asked whether RIG‐I directly binds viral RNAs. We employed an in vitro assay in which recombinant FLAG‐tagged RIG‐I protein and total RNA from infected cells were mixed in a test tube 16. After incubation, RIG‐I was immunoprecipitated with α‐FLAG antibodies and RNAs that co‐purify with the protein were extracted. These RNAs were then analyzed for their potential to induce a response in our *IFNβ* promoter reporter cells and for the presence of viral RNAs (Fig. 2A). Negative controls included immunoprecipitation with non‐specific control antibody and the use of denatured RIG‐I protein. Western blot analysis confirmed that both native and denatured RIG‐I were precipitated by α‐FLAG but not by control antibody (Fig. 2B, E and data not shown). As RIG‐I recognizes the RNA genome of IAV 31, we used total RNA from HEK293T cells infected with this virus as a positive control. As expected, native but not denatured RIG‐I bound to RNAs that stimulated reporter expression in a RIG‐I‐dependent manner (Fig. 2C and data not shown). RT‐qPCR analysis confirmed that RNA corresponding to the M segment of IAV was bound by native RIG‐I (Fig. 2D). Similarly, pulldown of native RIG‐I mixed with A549‐ZIKV‐RNA retained IFN‐stimulatory RNAs (Fig. 2F). Moreover, RT‐qPCR using a probe corresponding to nucleotides 4930–5029 of the full length ZIKV RNA genome demonstrated an association of ZIKV RNA with RIG‐I (Fig. 2G). This finding is consistent with the accumulation of RIG‐I‐stimulatory RNAs in ZIKV‐infected cells and shows that RIG‐I is capable of recognizing ZIKV RNAs.

### Construction of ZIKV Brazil protein expression vectors

**Figure 3:**
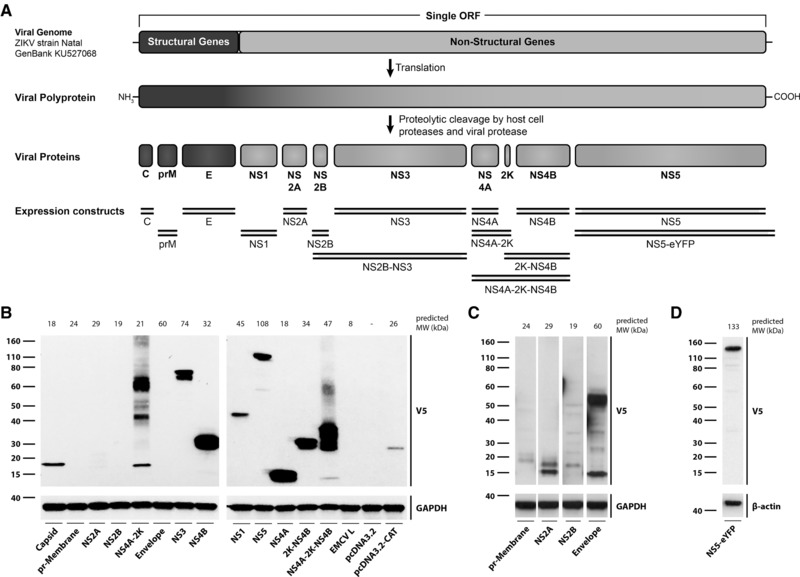
Cloning and expression of ZIKV proteins. (A) Expression constructs. The DNA sequence of the single ZIKV open reading frame encoding all structural and non‐structural proteins was derived from a sequenced ZIKV complete genome (GenBank accession KU527068; origin Brazil) recovered from the brain of a microcephalic, aborted foetus. Segments encoding individual proteins were annotated using sequence alignment with a reference genome (GenBank accession NC_012532.1). Expression constructs generated and used in this study are shown as double lines. (B) HEK293 cells were transfected with plasmids expressing the indicated ZIKV proteins. Cell lysates were prepared 24 h after transfection and protein expression was determined by Western blot using the indicated antibodies with GAPDH as loading control. MW, molecular weight. (C, D) The experiment shown in panel (B) was repeated using HEK293T cells and the indicated plasmids. Data are representative of at least three independent experiments.

To further study the interaction between ZIKV and the type I IFN system, we prepared a library of expression constructs for individual ZIKV proteins. Importantly, we based our analysis on the sequence of ZIKV strain Natal RGN, Brazil (KU527068) 11. In contrast, an earlier study used sequences derived from a viral strain from French Polynesia 19. In brief, coding sequences were assembled by gene synthesis, cloned into a gateway entry vector and then shuttled into pcDNA3.2 for expression in mammalian cells with an C‐terminal V5 tag. Our library included the three structural proteins (C, prM, E), the seven non‐structural proteins (NS1, NS2A, NS2B, NS3, NS4A, NS4B, NS5), as well as several fusion proteins (NS2B‐NS3, NS4A‐2K, 2K‐NS4B, NS4A‐2K‐NS4B) (Fig. 3A). Following transient transfection of HEK293 cells, expression of ZIKV proteins was tested by Western blot using an antibody against the V5 tag (Fig. 3B–D). C, NS1, NS3, NS4A, NS4B, NS5 and 2K‐NS4B constructs were expressed well and a single band of the predicted molecular weight was detected (Fig. 3B). Other constructs were expressed at lower levels and for some multiple bands were evident, which may reflect post‐translational modifications, protein degradation or aggregation (Fig. 3B, C). We also constructed a vector expressing NS5 fused at its C‐terminus to enhanced yellow fluorescent protein (eYFP) for localization studies (Fig. 3D).

### ZIKV NS5 inhibits the induction of type I IFN downstream of RLRs

**Figure 4:**
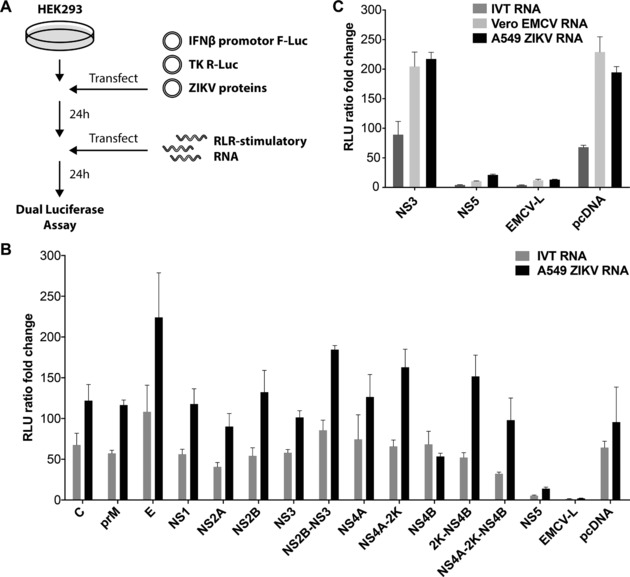
ZIKV NS5 blocks type I IFN induction by RLRs. (A) Schematic of the experimental setup. Please see results for detail. (B, C) HEK293 cells were transiently transfected with the indicated ZIKV plasmids, a plasmid encoding F‐Luc under control of the *IFNβ* promoter and a plasmid expressing *Renilla* luciferase (R‐Luc) from the constitutive thymidine kinase (TK) promoter. Cells were transfected with RLR‐stimulatory RNAs after 24 h as indicated. F‐Luc and R‐Luc activities in cell lysates were determined after an additional 24 h and the F‐Luc / R‐Luc ratio was set to 1 in cells that received only transfection reagent at the RNA transfection step. Data are representative of three independent experiments (average and SD (*n* = 3)).

Given that ZIKV infection generates RNAs that activate the RLR pathway (Figs. 1 and 2), we asked whether the virus encodes an antagonist of RLR‐driven type I IFN induction. To test this idea, we transiently transfected HEK293 cells with ZIKV expression vectors. We included an *IFNβ* promoter F‐Luc reporter plasmid and, for normalization, a *Renilla* luciferase control plasmid in the transfection mix. After overnight incubation, cells were stimulated with RLR agonists and induction of the reporter was determined (Fig. 4A). Empty vector and an expression plasmid for EMCV‐L, a known antagonist of IRF3 35, served as negative and positive controls, respectively. As expected, *IFNβ* promoter activation by the synthetic RIG‐I agonist IVT‐RNA and by A549‐ZIKV‐RNA were blunted by EMCV‐L expression (Fig. 4B). Interestingly, ZIKA NS5 had similar inhibitory effects, while the other viral proteins had no effect on expression of the *IFNβ* promoter reporter (Fig. 4B). NS5 also blocked activation of the reporter by an MDA5 agonist, VERO‐EMCV‐RNA 32 (Fig. 4C). These data indicate that ZIKV NS5 is a viral inhibitor of type I IFN induction mediated by RLRs.

### NS5 blocks type I interferon receptor signaling

**Figure 5:**
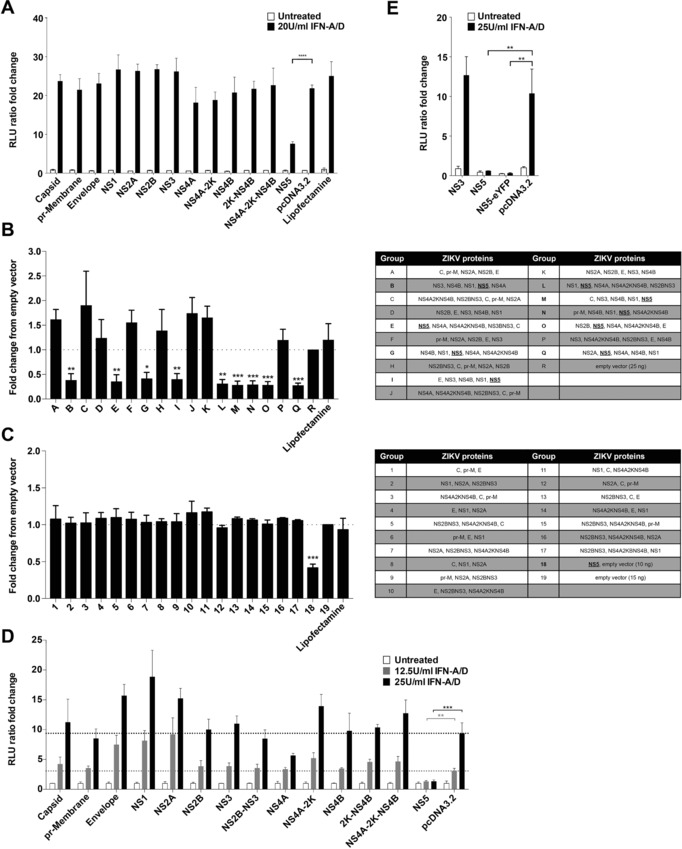
ZIKV NS5 blocks type I IFN signaling. (A) Stable HEK293‐ISRE‐reporter cells were transfected with ZIKV plasmids as indicated and, 24 h later, were treated or were not with 20U/ml IFN‐A/D. Luciferase activity in cell lysates was determined 24 h after transfection and was set to 1 in untreated cells that received empty vector. (B,C) The experiment shown in (A) was repeated using plasmid combinations as indicated on the left. (D,E) HEK293T cells were transiently transfected with the indicated ZIKV plasmids, a plasmid encoding F‐Luc under ISRE control and a plasmid expressing *Renilla* luciferase (R‐Luc) from the TK promoter. Cells were treated with IFN‐A/D after 24 h as indicated. F‐Luc and R‐Luc activities in cell lysates were determined after an additional 24 h and the F‐Luc / R‐Luc ratio was set to 1 in untreated cells that received empty vector. Panels (A), (D), (E) are representative of three independent experiments (average and SD (*n* = 3)). Panels (B) and (C) show pooled data from three experiments (average and SEM (*n* = 9)). (A), (D), (E): unpaired, two‐tailed *t*‐test as indicated. (B), (C): unpaired, two‐tailed t‐test vs empty vector. (^*^ *p*<0.05, ^**^ *p*<0.01, ^***^ *p*<0.001, ^****^ *p*<0.0001).

We next investigated whether NS5 or other ZIKV proteins block type I interferon receptor signaling. We recently described a stable reporter cell line that contains F‐Luc under the control of an interferon‐stimulated response element (ISRE) 36. Exposure to type I IFN induces F‐Luc expression in these cells. We tested whether overexpression of ZIKV proteins blocks the induction of the ISRE reporter gene. When ZIKV proteins were expressed individually, only NS5 reduced F‐Luc induction in response to recombinant type I IFN (Fig. 5A). Other ZIKV proteins did not reduce the induction of the ISRE reporter (Fig. 5A). It is possible that different ZIKV proteins may act together to block IFNAR signaling; hence, we repeated the experiment by co‐transfecting groups of five ZIKV expression plasmids arranged so that each ZIKV protein was expressed with every other protein at least once. Any plasmid combination containing ZIKV NS5 blocked ISRE induction, while combinations not including NS5 did not interfere with the response to type I IFN (Fig. 5B). In line with this observation, we found that combinations of three plasmids not including NS5 did not block IFNAR signaling (Fig. 5C).

The effect of NS5 in the stable reporter cells used in Figs. 5A–C was incomplete. This may be due to limited transfection efficiency and responses from non‐transfected cells contained in the well. We therefore repeated the experiment by transient transfection of an ISRE‐F‐Luc reporter plasmid together with ZIKV expression constructs and a constitutively expressed *Renilla* luciferase plasmid. In this setting, the response to recombinant type I IFN was completely blocked by NS5 (Fig. 5D). NS4A weakly blocked ISRE induction and all other ZIKV proteins failed to have an effect (Fig. 5D). N‐terminally eYFP tagged NS5 also blocked ISRE induction (Fig. 5E).

**Figure 6:**
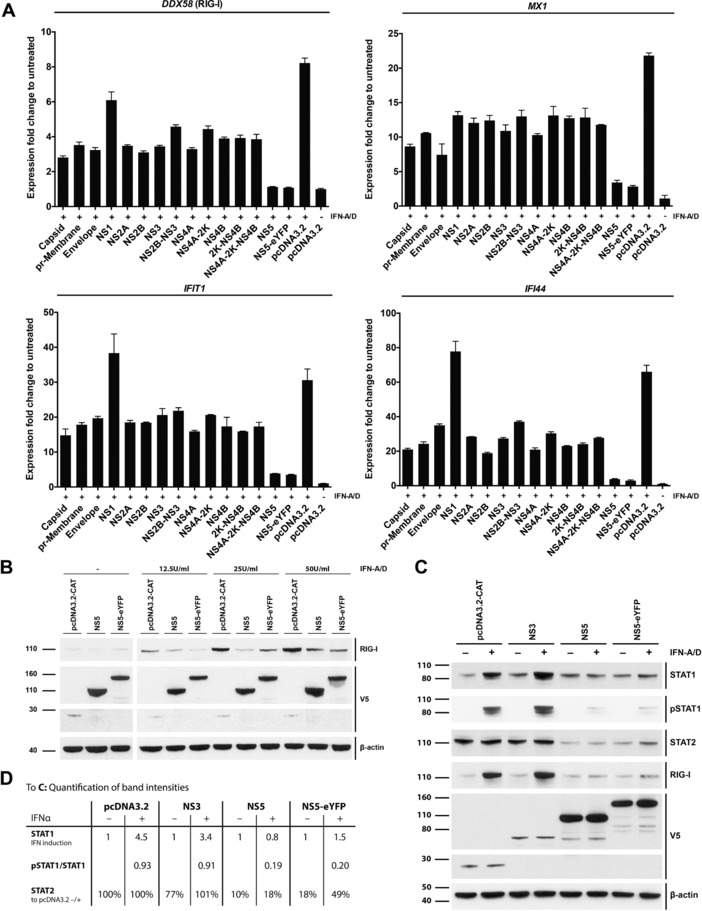
NS5 triggers STAT2 depletion and blocks STAT1 phosphorylation. (A) HEK293T cells were transfected with the indicated ZIKV plasmids and either were or were not treated with 25U/ml of IFN‐A/D. RNA was extracted after 24 h and expression levels of the indicated mRNAs were determined by RT‐qPCR with samples assayed in technical duplicate. Data were analyzed by the comparative C_t_ method and set to 1 for untreated cells transfected with empty vector. (B,C) Cell lysates were prepared from HEK293T cells transfected with the indicated plasmids and treated with IFN‐A/D for 24 h. Expression of the indicated protein was determined by Western blot with β‐actin as loading control. (D) Quantification of signals in (C) using the image densitometry analysis tool of ImageJ. Panels (A) ‐ (C) are representative of three independent experiments. Panel (A) shows average and range (*n* = 2).

To test whether NS5 interferes with induction of endogenous ISGs after exposure of cells to type I IFN, we measured the expression of *DDX58* (encoding RIG‐I), *MX1*, *IFIT1* and *IFI44* by RT‐qPCR. Expression of these ISG mRNAs was induced to different levels after type I IFN treatment and this was blocked by the presence of NS5 (Fig. 6A). Similarly, RIG‐I protein levels were induced by type I IFN and this was prevented by NS5 expression (Fig. 6B). Taken together, these data demonstrate that NS5 is the major antagonist of IFNAR signaling and ISG induction encoded by a ZIKV isolate from Brazil.

### ZIKV NS5 depletes STAT2 levels and blocks STAT1 phosphorylation

Earlier work suggested that ZIKV NS5 targets STAT2 for degradation 19, 28 and that ZIKV infection blocks STAT1 phosphorylation 27. Therefore, we investigated the mechanism by which NS5 interferes with IFNAR signaling and tested STAT1 and STAT2 expression and phosphorylation levels by Western blot. NS5 bound to STAT2 and STAT2 levels were reduced by NS5 suggesting that NS5 may target STAT2 for degradation (Fig. 6C and S2). In contrast, STAT1 protein levels were unchanged in NS5 expressing cells that were not treated with IFN (Fig. 6C). *STAT1* is an ISG 17 and, as expected, induction of STAT1 protein by IFN was blocked by NS5 (Fig. 6C). Moreover, STAT1 phosphorylation was readily detectable in control cells treated with type I IFN but was strongly reduced in NS5 expressing cells (Fig. 6C). Analysis of the ratio of total and phosphorylated STAT1 by densitometry showed that NS5 expression prevented STAT1 phosphorylation (Fig. 6D) suggesting that NS5 is the viral protein blocking STAT1 phosphorylation during ZIKV infection 27. Taken together, these data indicate that NS5 blocks IFNAR signaling by multiple mechanisms, including reduced STAT2 levels and inhibition of STAT1 phosphorylation.

### NS5 nuclear localization is not required for inhibition of IFNAR signaling

**Figure 7:**
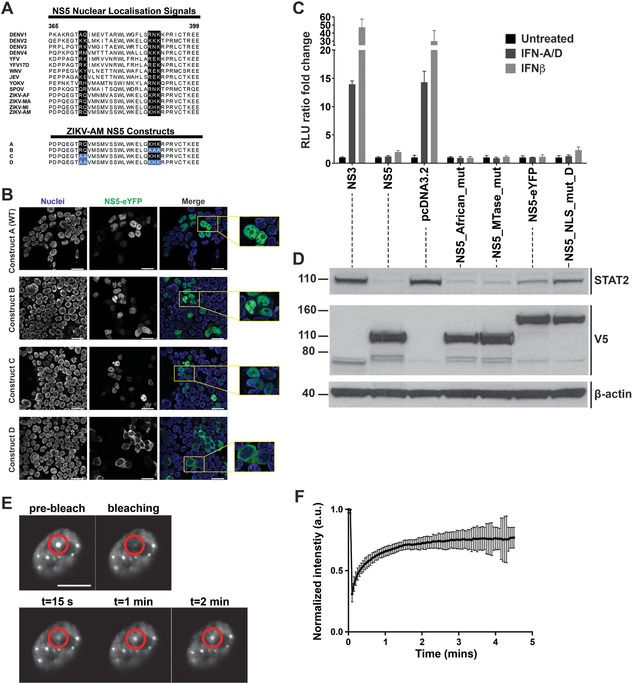
Blockade of type I IFN signaling by NS5 does not require NS5 nuclear localisation. (A) The NS5 interdomain regions from different flaviviruses were aligned and the area around the aNLS is shown. Residues involved in nuclear localization of DENV NS5 are highlighted in black. The aNLS mutations studied in (B) are indicated in blue at the bottom. DENV: Dengue virus; YFV: yellow fever virus; WNV: West Nile virus; JEV: Japanese encephalitis virus; YOKV: Yokose virus; SPOV: Spondweni virus; ZIKV: Zika virus – AF: Africa, MA: Malaysia, MI: Micronesia, AM: America. (B) HEK293T cells were transfected with wild‐type NS5‐eYFP or NS5 NLS mutant constructs as shown in panel (A). After 24 h, live cells were imaged with a confocal microscope. Nuclei were stained with the membrane permeable dye, NucBlue™. Scale bar is 25 μm. (C, D) HEK293T cells were transfected as in Fig. 5D using the indicated ZIKV plasmids and were treated with 25U/ml of IFN‐A/D or IFNβ. 24 h after transfection, luciferase activities were analyzed as in Fig. 5D (C) and cell lysates from untreated samples were analyzed by Western blot with β‐actin as loading control (D). (E) HEK293T cells were transfected with ZIKV NS5‐eYFP. After 24 h, the mobility of nuclear NS5 speckles was analyzed by fluorescence recovery after photobleaching (FRAP). Representative snapshots of a cell nucleus expressing NS5‐eYFP before, during and after bleaching of the speckle indicated by the red circle are shown, indicating that fluorescence of NS5 recovered and that the protein was mobile and in a fluid environment. Scale bar is 2 μm. (F) The intensity of NS5 speckles was normalized to 1 before photobleaching and was analyzed over time after photobleaching. The fluorescence intensity in NS5 speckles dropped to ∼25% of the initial value and then recovered back to ∼75%. Panels (B)–(F) are representative of at least three independent experiments. Panel C shows average and SD (*n* = 3). Panel (F) shows average of five speckles and SD.

ZIKV NS5 is predominantly localized in the cell nucleus and binds to importin α/β1 28, 37, 38, 39. NS5 has an N‐terminal methyltransferase (MTase) domain and a C‐terminal RNA polymerase domain, which are separated by the interdomain region. In DENV NS5, this region contains nuclear localization signals (NLSs) including the aNLS element that is recognized by importin α/β1 40, 41. The DENV NS5 aNLS is characterized by two clusters of functionally essential basic residues 41, some of which are conserved in ZIKV (Fig. 7A). To test if these residues are required for nuclear localization of ZIKV NS5, we mutated both clusters individually and together (Fig. 7A). We then expressed wild‐type and mutant NS5‐eYFP fusion proteins in HEK293T cells and analyzed NS5 localization by fluorescence microscopy. As expected, wild‐type NS5 was found in the cell nucleus (Fig. 7B). Mutation of cluster 1 (RQ to AA) or cluster 2 (KHK to AAA) did not change this pattern, while mutation of both clusters together resulted in redistribution of NS5 to the cytoplasm (Fig. 7B). This observation defines the RQ‐KHK aNLS motif in ZIKV NS5 as a functional bipartite NLS. Interestingly, we found that the NS5 NLS mutant was capable of blocking ISRE induction and reducing STAT2 levels (Fig. 7C, D).

The MTase activity of NS5 contributes to the formation of cap structures on viral transcripts by guanine N‐7 and ribose 2’‐OH methylation 42. The E218A mutation in yellow fever virus NS5 disrupts ribose 2’‐OH methylation 43. To test if MTase activity, in the absence of the canonical viral RNA substrate, is related to the effects of NS5 on IFNAR signaling, we introduced the same mutation into ZIKV NS5. However, overexpression of the E218A NS5 mutant in the absence of a viral substrate for methylation neither impaired NS5‐mediated inhibition of ISRE induction nor STAT2 depletion (Fig. 7C, D). Comparison of the African MR‐766 ZIKV isolate and Brazilian strains revealed two amino acid substitutions in NS5, M2634V in the MTase domain and M3392V in the RNA polymerase domain 3. We reverted these two valines at position 2634 and 3392 in our NS5 construct to methionine to test if these residues are involved in antagonism of IFNAR signaling. However, this “NS5 African mutant” behaved like the parental NS5 construct based on the Brazilian strain sequence (Fig. 7C and D). Taken together, these data show that NS5 nuclear localization was not required for blocking IFNAR signaling.

Our analysis of ZIKV NS5 localization showed that NS5 is not uniformly distributed across the cell nucleus, but appears to be enriched in dot‐like structures (Fig. 7B). We investigated the dynamics of these NS5 speckles using fluorescence recovery after photobleaching (FRAP). NS5 speckles recovered at the site of bleaching within about 1 min, suggesting that these structures are highly dynamic and presumably “liquid” in nature 44 (Fig. 7E and F).

## Discussion

Innate immune responses play an important role in determining the course of disease caused by virus infection. It is therefore important to understand how such responses are initiated and how viruses evade and antagonize them. Here, we studied this question in the context of the emerging pathogen ZIKV, and show that ZIKV infection generates RNA molecules that stimulate RIG‐I and MDA5. These sensors of virus invasion induce type I IFNs, which activate an anti‐viral gene expression program by signaling through their receptor, IFNAR. We report that ZIKV antagonizes RLR and IFNAR signaling by means of its NS5 protein, and investigate sequences and cellular location responsible.

Our results demonstrate that ZIKV infection results in the accumulation of RIG‐I stimulatory RNAs. RIG‐I detects RNA molecules bearing 5’‐triphosphate groups 31. NS5 is an RNA‐dependent RNA polymerase that replicates the genomes of viruses belonging to the *Flaviviridae* family and employs a primer‐independent mechanism of initiation 45. This results in the presence of triphosphate groups derived from the initiating ribonucleoside triphosphate at the 5’‐ends of nascent viral RNAs, which we predict activate RIG‐I in ZIKV infected cells. Indeed, we report that RIG‐I could directly associate with ZIKV RNA and that phosphatase‐treatment renders RNA from ZIKV infected cells unable to stimulate RIG‐I. It will be interesting to confirm this by infection experiments in RIG‐I‐deficient cells and by RIG‐I pulldowns from infected cells.

Flaviviral replication complexes assemble on ER membranes and reside in vesicles formed by membrane invaginations, also known as replication factories 45. How then, does RIG‐I–‐which is localized in the cytosol‐–gain access to newly replicated viral RNAs? Analysis of ZIKV infected cells by electron microscopy revealed pore‐like openings connecting replication factories to the cytosol 46. It is conceivable that at least some nascent viral RNAs escape through these pores and are then sensed by RIG‐I. Indeed, the RIG‐I pathway is very sensitive and is triggered by less than 20 RNA molecules per cell 47. Moreover, replication factories are likely to be dynamic structures.

Interestingly, we find that ZIKV infection generates not only RIG‐I agonists but also MDA5‐stimulatory RNAs. Dual activation of RIG‐I and MDA5 is consistent with the observation that IFN induction downstream of both RIG‐I and MDA5 is blocked by sfRNA, a subgenomic, non‐coding RNA expressed during ZIKV infection 34. Furthermore, infection with other members of the *Flaviviridae* family, such as hepatitis C virus, activates both RLRs 48. Although the molecular properties of RNAs that activate MDA5 are not fully understood, it is clear that MDA5 activation, in contrast to RIG‐I, does not require the presence of 5’‐triphosphates 13, 14. NS5 in conjunction with NS3 caps flaviviral RNAs by sequential triphosphatase, guanylyltransferase and methyltransferase activities 45. It is likely that capping is an evasion strategy that allows viral RNAs to escape detection by RIG‐I and other factors such as IFIT proteins 15. It is possible that MDA5 detects mature ZIKV RNAs and thereby functions as a backup if RIG‐I activation is limited by capping.

Many viruses, including flaviviruses, encode inhibitors of the type I IFN system 49, 50, 51. Viral antagonists typically dampen but do not completely shut‐down the host response and block type I IFN induction or repress IFNAR signaling. We found that ZIKV NS5 acted at both of these levels and prevented RLR‐driven type I IFN induction as well as IFN‐mediated ISG expression. The former observation suggests that the reduced type I IFN response reported in ZIKV infected cells transfected with synthetic RLR agonists 19, 27, 52 is due to NS5. The finding that type I IFN responses to both RIG‐I and MDA5 stimuli were blocked by NS5 further suggests that NS5 acts at the level of the common adaptor MAVS or downstream in the RLR signaling pathway. This is consistent with a recent study suggesting that NS5 interacts with and blocks IRF3 30. Another possibility is that NS5 inhibits RLR signaling indirectly by antagonizing ISG expression, as, in a feed‐forward loop, both RIG‐I and MDA5 are encoded by ISGs. It will be interesting for future studies to investigate the mechanism by which NS5 blocks RLR signaling.

Recent work using African and French Polynesian ZIKV isolates already demonstrated that ZIKV NS5 interferes with IFNAR signaling by targeting STAT2 for proteasomal degradation 19, 28. Here, we extend these findings in several ways. First, we show that NS5 from a Brazilian ZIKV strain collected from an aborted, microcephalic fetus blocks the response to type I IFN. This demonstrates that IFNAR signaling is targeted by a ZIKV isolate derived from the recent epidemic. Second, by using our expression constructs for ZIKV proteins alone or in combination, we report that NS5 is likely to be the only potent ZIKV antagonist of IFNAR signaling. It is possible that other viral proteins make a small contribution to blocking IFNAR signaling. For example, we found that NS4A expression slightly dampens the response to type I IFN (Fig. 5D) and Wu et al. as well as Xia et al. recently reported effects of similar magnitude for NS2B‐NS3 and other viral proteins 30, 53. It is likely that this fine‐tuning of the response depends on the virus strain, kinetics and precise experimental setup, while it is clear from our data and those of others 19, 28 that NS5 has the most potent and lasting effect. Thirdly, we show that ZIKV NS5 not only reduces STAT2 levels but also blocks STAT1 phosphorylation. This is consistent with a report studying the effects of ZIKV infection in dendritic cells 27 and indicates that ZIKV NS5 has evolved multiple strategies to counteract IFNAR signaling 50, 51. Fourthly, we define a NLS in ZIKV NS5. Earlier work using the African MR‐766 strain found that deletion of four amino acids (KRKR), which overlap with the second half of the bipartite RQ‐KHK motif identified here (Fig. 7A), abrogates nuclear localization of overexpressed NS5 38. In contrast, we found that the KHK residues in NS5 from a Brazilian isolate were required, but not sufficient, for nuclear localization of the protein, which also required the RQ motif. These observations highlight differences between ZIKV strains. Furthermore, we demonstrate that NS5 nuclear localization was not required for the inhibition of IFNAR signaling. Future studies should address the molecular mechanism by which NS5 targets STAT transcription factors. For example, it will be interesting to identify the E3 ubiquitin ligase required for STAT2 degradation.

It will also be important to delineate the role of NS5 in the cell nucleus. It has been suggested that nuclear NS5 from other flaviviruses blocks IL‐8 induction [reviewed in: 54]. Interestingly, we found that NS5 is organized as speckles in the nucleus. Several recent studies reported similar findings for overexpressed and endogenous NS5 28, 38, 39. Using immunofluorescence with α‐PML antibody, we found that these structures do not correspond to PML bodies (data not shown). Instead, it is possible that these nuclear NS5 speckles correspond to sites of splicing, given that NS5 co‐localizes with SC35, a cellular protein involved in pre‐mRNA splicing 38. Indeed, DENV NS5 has been reported to bind to components of the spliceosome and to modulate splicing of host genes, including *DDX58* (encoding RIG‐I) 55. We further report that the molecules in these speckles were in constant exchange with the surrounding NS5, which suggests that these compartments are “liquid” in nature. This kind of liquid‐liquid phase separation has been described for proteins forming membrane‐less compartments such as P granules, typically together with nucleic acids or other proteins 56, 57. This type of compartmentalization plays a role in several processes such as RNA metabolism, biogenesis of the ribosome, DNA damage response and signal transduction 58. Therefore, it will be an intriguing quest for the future to elucidate the role of this compartmentalization in NS5 function.

Finally, it would be interesting to define mutations in NS5 that selectively disrupt its ability to block RLR or IFNAR signaling while leaving intact its essential functions in the viral life cycle. We predict that such mutant viruses induce elevated IFN responses and only replicate efficiently in cells deficient for components of the type IFN system.

Taken together, we define the interplay between ZIKV and the type I interferon system and identify three points of interaction between the virus and cells: ZIKV infection generates stimuli for the pattern‐recognition receptors RIG‐I and MDA5, and the viral NS5 protein blocks the ensuing antiviral response by antagonizing RLR signaling and STAT transcription factors that are involved in IFNAR signaling. It is likely that these host‐pathogen interactions are important for determining the outcome of disease.

## Materials and methods

### Cells

Cells were maintained in DMEM (Sigma Aldrich) supplemented with 10% FCS (Sigma‐Aldrich) and 2 mM L‐Glutamine (Gibco) at 37°C and 5% CO_2_. 3C11 cells are HEK293 cells (originally from Caetano Reis e Sousa, The Francis Crick Institute, London, UK) stably transduced with an ISRE‐Luc reporter construct 36. A549 cells were obtained from R. E. Randall, University of St Andrews, UK.

### Plasmids

To generate p125‐GreenFire, the human *IFNβ* promoter region was amplified from the p125‐Luc plasmid 59 and cloned into the pGreenFire1‐ISRE lentiviral reporter system (System Biosciences, TR016PA‐P) using EcoRI and BamHI restriction enzymes to excise the ISRE elements and the minimal CMV promoter. The puromycin N‐acetyl‐transferase gene was replaced by the blasticidin‐S deaminase gene from the pLenti6.3/V5‐DEST™ Gateway® Vector (Invitrogen) using the restriction enzyme KpnI.

Plasmids used for luciferase reporter assays were p125‐Luc 59, ISG54‐Luc (Clontech), and pRL‐TK (Promega).

A DNA sequence of the single ZIKV open reading frame encoding for all structural and non‐structural proteins was derived from a sequenced ZIKV complete genome (GenBank accession KU527068) recovered from the brain of a microcephalic aborted foetus 11. The genome was annotated for sequence segments encoding individual proteins using sequence alignment with an annotated reference genome (GenBank accession NC_012532.1) 60. DNA fragments encoding for the Capsid, Envelope, and pr‐Membrane proteins as well as the non‐structural proteins NS2A, NS2B, NS3, NS4A‐2K, and NS4B were obtained by gene synthesis (GeneArt String linear DNA fragments; Thermo Fisher Scientific) after addition of a start codon (AUG) to the 5' end of DNA sequences. Fragments were cloned into the pCR8/GW/TOPO Gateway entry vector (Invitrogen). After sequence integrity was verified *via* sequencing, inserts were Gateway‐cloned into the pcDNA3.2/V5‐DEST vector (Invitrogen) using the Gateway LR Clonase II Plus Enzyme Mix (Invitrogen). Fragments encoding the non‐structural proteins NS1 and NS5 were generated by gene synthesis into the pDONR221 Gateway donor vector (GeneArt Gene, Thermo Fischer Scientific) and Gateway‐cloned into the pcDNA3.2/V5‐DEST vector. DNA constructs encoding the other ZIKV proteins utilized in this project (NS4A, 2K‐NS4B, NS4A‐2K‐NS4B, NS2B‐NS3, NS5‐eYFP) were created by (Overlap)‐PCR from existing ZIKV protein encoding plasmids using Phusion High Fidelity DNA Polymerase (New England Biolabs). NS5 mutants were created by mutagenesis PCR and confirmed by sequencing. ZIKV expression plasmids will be made available via Addgene.

### A549‐ZIKV‐RNA, IVT‐RNA, VERO‐EMCV‐RNA and HEK293T‐IAV‐RNA

A549‐ZIKV‐RNA was generated by infection of A549 cells with ZIKV (isolate ZIKV/H.sapiens/Brazil/PE243/2015, GenBank accession KX197192.1, described in 34) at an MOI of 5. Unless indicated otherwise, cells were lysed in TRIzol (Invitrogen) 20 h later and total RNA was extracted according to manufacturer's instructions. Neo^1‐99^ in vitro transcribed RNA was generated as previously described 31. Purified RNAs were incubated with alkaline phosphatase (Roche) using 2 units of enzyme per microgram of RNA. Samples were incubated at 50°C for 1 h and RNAs were purified by phenol‐chloroform extraction. Negative control reactions were performed in parallel by omitting the enzyme. VERO‐EMCV‐RNA was generated as described previously 32. VERO EMCV‐RNA (CIP) was created by treatment of purified Vero EMCV‐RNA with 2 units calf intestinal phosphatase (CIP) (Roche) per μg of RNA for 1 h at 50°C and subsequent purification by phenol‐chloroform extraction. HEK293T‐IAV‐RNA was generated by infection of HEK293T cells with influenza A virus (IAV) (A/PR/8/34 (H1N1), kind gift from Alain Townsend, Oxford, UK) at an MOI of 5. Total RNA was extracted 24h later using TRIzol according to manufacturer's instructions.

### Generation and validation of the p125‐HEK reporter cell line

Lentiviruses were produced in HEK293T cells (kind gift from Caetano Reis e Sousa, The Francis Crick Institute, London, UK), using Fugene6 (Promega) to transfect the p125‐GreenFire plasmid in combination with the Vira Power packaging mix (Invitrogen). Medium was replaced the day after transfection. Supernatant was collected 48 h post transfection and filtered through a 0.45 μm PES filter. 1.5 × 10^6^ HEK293 cells (kind gift from Caetano Reis e Sousa, The Francis Crick Institute, London, UK) were subsequently infected with 1 mL of viral supernatant in the presence of 8 μg/mL of polybrene. The following day, the inoculum was removed and fresh medium was added to the cells. One week later, cells were cloned by limiting dilution. After 26 days, several clones were harvested from plates that had initially been seeded with less than 0.4 cells per well. These clones were expanded and tested by transfection of IVT‐RNA. Clone 17 was selected for having both, a high amplitude of response and a high sensitivity.

### Generation and validation of p125‐HEK KO cell lines using CRISPR technology

sgRNAs targeting RIG‐I, MDA5 and MAVS were selected based on the MIT algorithm (crispr.mit.edu). Specifically, the RIG‐I sgRNA targets exon 2, the MDA5 sgRNA exon 1, and the MAVS sgRNA exon 5, which is the first common exon shared by the full‐length protein and Mini‐MAVS 61. All sgRNAs were cloned into a modified version of the pX458 plasmid (Addgene 48138, deposited by Dr. Feng Zhang). We replaced the eGFP moiety of pX458 by insertion of the mRuby2 cDNA and all sgRNAs were cloned using the dual BbsI strategy and the incompatible CACC/AAAC overhangs (oligos used for cloning are listed in Supporting Information Table 1). For RIG‐I, an additional G was added at sgRNA position 0 to reconstitute an optimum Pol‐III promoter, whereas for MDA5 and MAVS the original protospacers were cloned.

For the generation of the KO cell lines, p125‐HEK clone 17 cells were seeded in a 6‐well plate at 8.5 × 10^5^ cells per well one day before transfection with 5 μg of plasmid complexed with 5 μL of Lipofectamine LTX (Invitrogen) and 5 μL of Plus reagent per well. The medium was replaced one day later. On the following day, cells were harvested and single Ruby‐positive cells were FACS‐sorted into 96‐wells. Clones were harvested 27 days later.

In order to identify the mutations at the target loci, sgRNA targeted p125‐HEK cells were washed with PBS and re‐suspended in QuickExtract solution (Epicentre, catalogue # QE 09050). After 15 seconds of vortexing, lysates were incubated for 6 min at 65°C, vortexed again, then incubated for 2 min at 98°C. 200ng of DNA was used in a 50 μL PCR reaction using Phusion polymerase in the presence of 3% of DMSO. PCR products were gel purified with QIAquick Gel Extraction Kit and sequenced using the PCR primers listed in Supporting Information Table 1. Sequences were analyzed using the TIDE software (https://tide.nki.nl) 62.

To further validate the KO cell lines, cells were seeded into either 24‐well (WT, RIG‐I KO and MAVS KO) or 6‐well (WT and MDA5 KO) plates. Once almost confluent, cells were either treated with 140U/mL of IFN‐A/D overnight before lysis in 1% NP‐40 lysis buffer (25 mM Tris‐HCl pH7.4, 150 mM NaCl, 2 mM EDTA, 5% glycerol, 1% NP‐40, 1:100 Protease inhibitor cocktail) or infected with Sendai virus at an MOI of 0.5 per well for 24 h, before lysis in 0.5% NP‐40 lysis buffer (20mM Tris‐HCl pH7.4, 100 mM NaCl, 1 mM EDTA, 0.5% NP‐40, 1:100 Protease inhibitor). Lysates were incubated for 30 min on ice, then cellular debris was removed by centrifugation and supernatants were used for SDS‐PAGE and blotting as described below.

### p125‐HEK cell stimulation

p125‐HEK clone 17 cells were seeded at 50 000 cells per well in a 96‐well plate. On the following day, cells were transfected with different doses of IVT‐RNA or A549‐ZIKV‐RNA complexed with 0.2 μL of Lipofectamine per well. Activation of the *IFNβ* promoter was assessed one day later by OneGlo luciferase assay (Promega).

RIG‐I‐ and MDA5‐KO p125‐HEK cells were validated by stimulation with IVT‐RNA and VERO‐EMCV‐RNA (CIP), selective RIG‐I and MDA5 agonists, respectively (Supporting Information Fig. 1N). To validate that MAVS‐KO p125‐HEK cells can still activate the *IFNβ* promoter, WT and MAVS‐KO p125‐HEK cells were seeded at 20 000 cells per well in a 96‐well plate. On the next day, cells were treated with 200U/mL IFN‐α. After 24 h, cells were transfected with 100 ng of pcDNA3‐MAVS. Luciferase activity was measured 1 day later.

### FACS

We adhered to EJI guidelines for FACS. p125‐HEK cells were seeded at 200 000 cells per well in a 24‐well plate 1 day before transfection with graded doses of IVT‐RNA complexed with 1 μL of Lipofectamine. On the next day, cells were trypsinized, resuspended in FACS buffer (PBS, 1% FCS, 2mM EDTA, 0,02% sodium azide) containing 1 μg/mL of DAPI and immediately analyzed using a CyAn ADP High‐Performance Flow Cytometer (DakoCytomation). Cellular debris and doublets were gated out using Forward Scatter and Side Scatter Channels, and dead cells using DAPI staining (violet 1 channel). Between 27 000 and 30 000 live single cells were analyzed per sample for GFP expression.

### Antibodies

Primary antibodies utilized for immunoblotting were: STAT1 (clone 42H3, Cell Signaling, 1:1000), pSTAT1(Y701) (clone D4A7, Cell Signaling, 1:1000), STAT2 (clone D9J7L, Cell Signaling, 1:1000), RIG‐I (clone Alme‐1, AdipoGen, 1:1000), MAVS (Enzo Life Sciences #ALX‐210‐929‐C100, 1:500), MDA5 (clone 17, mouse monoclonal antibody raised in‐house), V5‐HRP (Invitrogen, 1:5000), beta‐actin‐HRP (clone AC‐15, Sigma Aldrich, 1:10 000), GAPDH‐HRP (Proteintech, 1:10 000) and FLAG‐HRP (clone M2, Sigma Aldrich, 1:10 000). HRP‐coupled secondary antibodies include sheep‐α‐mouse and donkey‐α‐rabbit (both GE Healthcare, 1:3000).

### Western blot analysis

HEK293 or HEK293T cells were seeded at a density of 8 × 10^5^ or 1 × 10^6^ cells/well, respectively, in 6‐well plates. 24 h later, cells were transfected with 1500 ng of ZIKV or control plasmids using Lipofectamine 2000 (Invitrogen). Where indicated cells were treated with recombinant human IFN‐A/D on day three (Sigma‐Aldrich or R&D Systems). After an additional 24 h incubation period, cells were lysed in lysis buffer (50 mM TRIS‐HCl pH 7.4, 150 mM NaCl, 1% Triton X‐100, 0.1% SDS, 0.5% sodium deoxycholate) containing 1:100 Protease Inhibitor Cocktail (Cell signaling Technology). Phosphatase Inhibitor Cocktail 3 (Sigma‐Aldrich) was added for analysis of STAT protein phosphorylation. Supernatants were removed after centrifugation. Protein concentrations were determined using Pierce BCA Protein Assay Kit (Thermo Scientific) and equalized by dilution of samples with lysis buffer. Subsequently, 5× Laemmli sample buffer (312.5mM TRIS‐HCl, 10% SDS, 25% beta‐mercaptoethanol, 50% glycerol, 0.01% bromophenol blue, pH 6.8) was added and samples were incubated at 95°C for 10 min. Samples were run on NuPAGE Novex 4–12% Bis‐TRIS gels (Invitrogen) using NuPage MOPS‐SDS running buffer (Invitrogen). Proteins were subsequently blotted onto PROTRAN Pure nitrocellulose membrane (PerkinElmer). Membranes were blocked with 5% skim milk powder (Sigma‐Aldrich) in TBS 0.1% Tween‐20 (5% milk TBS‐T) for 1 h at room temperature and were then incubated with primary antibodies in 5% milk TBS‐T overnight at 4°C. Primary antibodies that bind to phosphorylated residues were diluted in 5% BSA in TBS‐T. Membranes were washed thrice with TBS‐T and incubated with HRP‐coupled secondary antibodies in 5% milk TBS‐T for 1 h at room temperature. After three further washes with TBS‐T, proteins were detected using Western LightningPlus‐ECL (PerkinElmer) and Amersham Hyperfilm MP (GE Healthcare). If needed, antibodies were stripped from the membrane with stripping buffer (200 mM glycine, 0.1% SDS, 1% Tween‐20, pH 2.2) for 20 min at room temperature. Membranes were washed with TBS‐T, blocked as previously and re‐probed.

### Luciferase assay

For luciferase assays, cells either stably transduced with F‐Luc reporter constructs (3C11, p125‐HEK, p125‐HEK‐KOs) or transiently transfected with F‐Luc reporter and *Renilla* luciferase control plasmids were utilized. For stable cell lines, One‐GLO reagent and GLO‐Max luminometer (both Promega) were used to measure F‐Luc intensity. For cells transfected with firefly and *Renilla* luciferase plasmids, Dual‐Luciferase Assay System (Promega) was used according to manufacturer's instructions and F‐Luc activity was normalized to R‐Luc activity.

### RT‐qPCR

A549 cells were infected with ZIKV (see above) at an MOI of 5 and total RNA was extracted after 4, 8, 24, 48 and 72 h using TRIzol (Invitrogen) according to manufacturer's instructions. RNA concentration was measured with a Nanodrop system (Thermo Fischer Scientific). RNA was reverse transcribed into cDNA using SuperScript II Reverse Transcriptase (Invitrogen). The PCR reaction containing TaqMan Universal PCR Master Mix (Applied Biosystems) and TaqMan Primer/Probes (see below) was run on 7500 Fast Real time PCR System (Applied Biosystems) or QuantStudio 7 Flex Real‐Time PCR System (Thermo Fischer Scientific) with the following cycle conditions: 10 min 95°C, and 40 cycles of 15 s 95°C followed by 1 min 60°C. Gene expression was analyzed with the C_t_ method using *GAPDH* expression for normalization.

HEK293T cells were seeded at a density of 1.5 × 10^5^ cells/well in 24 well plates. 24 h later, cells were transfected with 300 ng of ZIKV or control plasmids using Lipofectamine 3000 (Invitrogen). On day three of the experiment, cells were treated with recombinant IFN‐A/D. After an additional 24‐h incubation period, RNA was extracted from cells using the QIAshredder (Qiagen) and RNeasy Mini Kit (Qiagen) according to manufacturer's instructions. RNA was used for RT‐qPCR as described above.

TaqMan primer probes used include *GAPDH* (Assay ID: Hs02758991_g1), *DDX58* (RIG‐I) (Assay ID: Hs01061436_m1), *IFIT1* (Assay ID: Hs03027069_s1), *MX1* (Assay ID: Hs00895608_m1), *IFI44* (Assay ID: Hs00951349_m1) and *IFNB1* (Assay ID: Hs02621180_s1).

### Microscopy

NS5‐eYFP was excited with a 488 nm laser and the emission was collected between 520 and 570 nm. For visualization of cellular localization of NS5 NLS mutants in living cells, nuclei were co‐stained with the membrane permeable NucBlue^®^ Live ReadyProbes^®^ Reagent (Thermo Fisher). For FRAP analysis, 1% laser power (∼100 μW) was used for imaging. A circular region of interest (ROI) was bleached using 75% of the laser power (∼10 mW). 100 frames were recorded after the bleaching with no time interval between the frames. A recovery curve was generated using the Stack T‐Function ‐ Intensity vs. Time Monitor plugin of ImageJ. A Zeiss 880 confocal microscope was used in all experiments.

### RIG‐I pulldowns

For investigation of RIG‐I bound RNA, 1.25 μg recombinant FLAG‐RIG‐I protein 16 and 5 μg infected cell total RNA (see above) were incubated under rotation at 4°C for 1 h in 250 μL IP buffer (20 mM TRIS‐HCl pH 7.4, 100 mM NaCl, 1 mM EDTA, 0.5% NP‐40, 0.05 U/mL RNasin Plus RNase inhibitor (Promega)). Where indicated, RIG‐I protein was denatured by heating to 95°C for 10 min prior to mixing with RNA. As input samples, 5 and 40 μL were removed for Western Blot analysis and RNA extraction, respectively. Samples were rotated as previously with 4 μg α‐FLAG antibody (clone M2, Sigma‐Aldrich) or mIgG1 isotype control antibody (clone MOPC‐31C, BD Pharmingen) for 2 h. Samples were added to 50 μl Protein G Dynabeads (Invitrogen) and rotated again for 2 h. Beads were separated from supernatant and 5 and 40 μL were removed from supernatant as unbound samples. Beads were resuspended in IP buffer and split 10–90% for Western Blot and RNA extraction, respectively.

For Western blotting, 4× Laemmli sample buffer (200 mM TRIS‐HCl, 8% SDS, 20% beta‐mercaptoethanol, 40% glycerol, 0.01% bromophenol blue, pH 6.8) was added to input, unbound and bound fractions and samples were incubated at 95°C for 10 min. Beads were removed and samples were subjected to SDS‐PAGE and blotting as described above.

TRIzol was added to input, unbound and bound fractions and RNA was extracted according to manufacturer's instructions. RNA was resuspended in 20 μL water. For luciferase assay, p125HEK reporter cells were seeded at 2.5 × 10^4^ cells/well in 96 well plates, transfected with 1 μL of RNA samples per well using Lipofectamine 2000 and assayed as described above. For RT‐qPCR, 4 μL of RNA samples were reverse transcribed into cDNA using SuperScript II Reverse Transcriptase (Invitrogen) in a 20 μL reaction. The 10 μL PCR reaction containing 1 μL cDNA, EXPRESS SYBR™ GreenER™ qPCR Supermix (Invitrogen) and 10 μM primers (see Supporting Information Table 1) was run on QuantStudio 7 Flex Real‐Time PCR System (Thermo Fischer Scientific) with the following cycle conditions: 10 min 95°C, and 40 cycles of 15 s 95°C followed by 1 min 60°C, and melt curve analysis. Abundance was analyzed with the C_t_ method and expressed as 2^−Ct^.

### Data analysis and statistics

Primary data were analyzed using Office Excel 2016 (Microsoft) and GraphPad Prism v7.00 (GraphPad Software). SnapGene (GSL Biotech) and ApE (M. Wayne Davis, The University of Utah) were utilized to assist cloning. The TIDE algorithm was used to analyze KO clone sequences 62. Quantitative analysis of Western blots was performed using ImageJ 63. Graphs and figures were created using GraphPad Prism v7.00 and Adobe Illustrator (Adobe Systems). Statistical analysis was performed in GraphPad Prism v7.00 as detailed in the Figure legends.

## Author contributions

A.G.D.J., J.H., R.E.R. and J.R. conceived the study, designed experiments, analyzed data and wrote the manuscript. A.G.D.J., J.H. and R.E.R. performed experiments. A.M. and P.H. generated and validated reporter and knockout cell lines. C.L.D. and A.K. provided RNA from ZIKV infected cells. E.S. performed FRAP experiments under supervision of C.E. C.S. and J.B. raised the MDA5 antibody. All authors read and approved the final manuscript.

## Financial support

This work was funded by the UK Medical Research Council [MRC core funding of the MRC Human Immunology Unit; J.R.], [MC_UU_12014, MR/N017552/1; A.K.] and by the Wellcome Trust [grant number 100954; J.R.]. A.G.D.J. is supported by CNPq [grant number 211806/2013‐7]. J.H. is supported by the European Commission under the Horizon2020 program H2020 MSCA‐ITN GA 675278 EDGE. The funders had no role in study design, data collection and analysis, decision to publish, or preparation of the manuscript.

## Conflict of interest

The authors declare no financial or commercial conflict of interest.

**Abbreviations**
DENV: Dengue virus
EMCV: Encephalomyocarditis virus
IFNs: Interferons
MAVS: Mitochondrial antiviral signaling protein
MOI: Multiplicity of infection
ZIKV: Zika virus

## Supporting information

Supplementary Information

Supplementary Information
