# Supplementary material for: Infection with a Brazilian isolate of Zika virus generates RIG‐I stimulatory RNA and the viral NS5 protein blocks type I IFN induction and signaling
Source: Eur J Immunol. 2018 Apr 6;48(7):1120–36. doi: 10.1002/eji.201847483 (PMC6055886; doi:10.1002/eji.201847483)
Supplement: Supplementary file 1 — Supplementary Information [file EJI-48-1120-s001.pdf]

European Journal of Immunology

Supporting Information
for
DOI 10.1002/eji.201847483

Jonny Hertzog, Antonio Gregorio Dias Junior, Rachel E. Rigby, Claire L. Donald, Alice Mayer, Erdinc Sezgin, Chaojun Song, Boquan Jin, Philip Hublitz, Christian Eggeling, Alain Kohl and Jan Rehwinkel

Infection with a Brazilian isolate of Zika virus generates RIG-I stimulatory RNA and the viral NS5 protein blocks type I IFN induction and signaling

© 2018 The Authors. *European Journal of Immunology* published by WILEY-VCH Verlag GmbH & Co. KGaA, Weinheim.

Supplementary Information

Infection with a Brazilian isolate of Zika virus generates RIG-I stimulatory RNA and the viral NS5 protein blocks type I IFN induction and signalling

Jonny Hertzog, Antonio Gregorio Dias Junior, Rachel E. Rigby, Claire L. Donald, Alice Mayer, Erdinc Sezgin, Chaojun Song, Boquan Jin, Philip Hublitz, Christian Eggeling, Alain Kohl and Jan Rehwinkel

Supplementary Figure 1

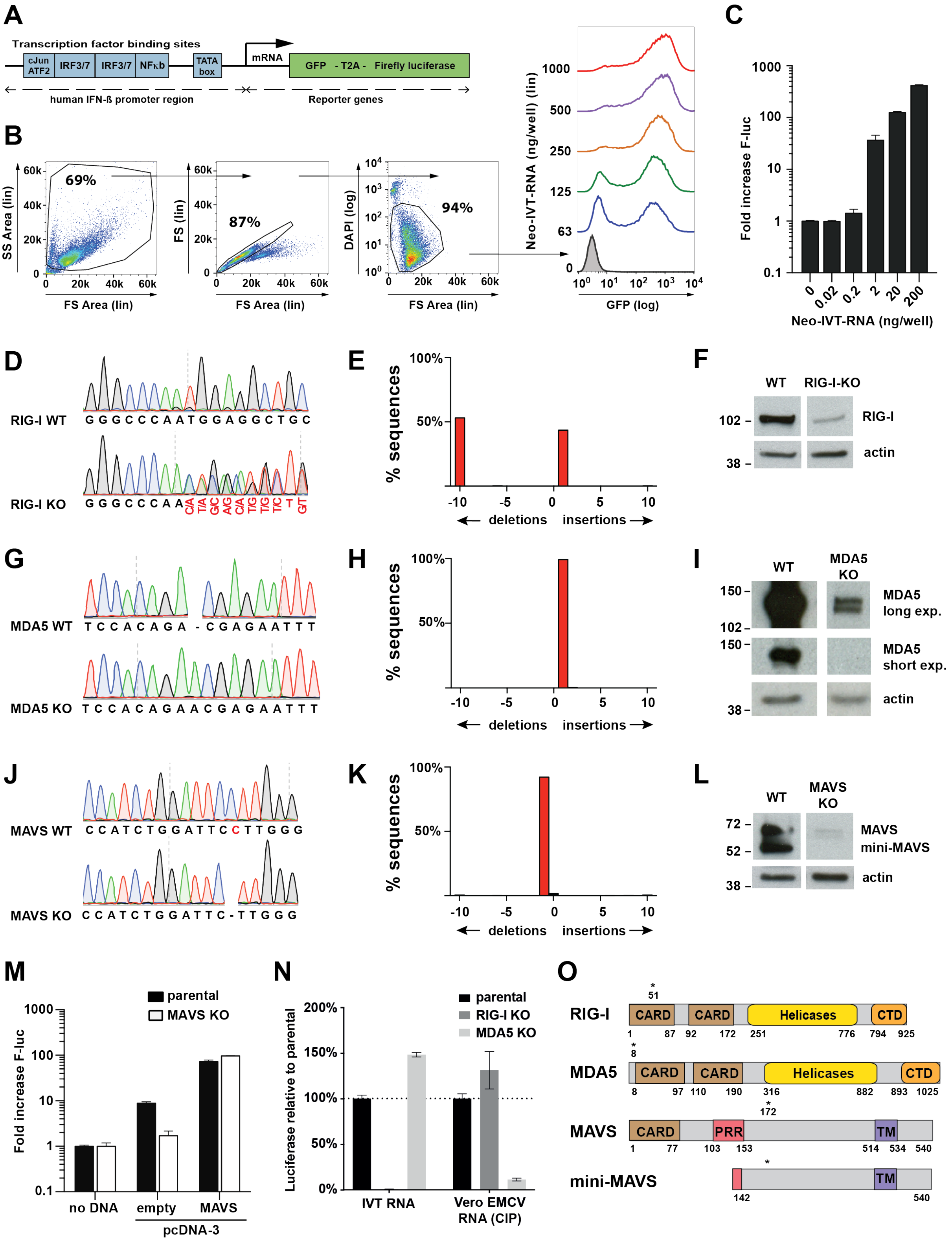

Fig. S1. Generation of RLR reporter cell lines.

(A) *IFNβ* reporter construct used to generate the reporter cell line. This construct co-expresses copGFP and F-Luc, linked by a self-cleaving T2A peptide, under the control of the human *IFNβ* promoter region.

(B) HEK cells stably transduced with the construct described in (A) - and hereafter referred to as p125-HEK cells - were seeded in a 24-well plate one day before transfection with graded doses of IVT-RNA. Cells were harvested 24 hours post transfection and expression of GFP was assessed by flow cytometry (gated on DAPI negative, single cells).

(C) p125-HEK cells were seeded in 96-well plates one day before transfection with graded doses of Neo^{1-99} IVT-RNA. F-Luc activity in cell lysates was determined 24 hours after transfection. Data are shown as fold change compared to cells treated with transfection reagent only.

(D - O) p125-HEK clones knocked out for RIG-I, MDA-5 and MAVS were generated using CRISPR technology.

(D, G, J) Genomic DNA was extracted from RIG-I KO (D), MDA5 KO (G) and MAVS KO (J) p125-HEK cells and from parental cells. A fragment of DNA surrounding the targeted area was amplified by PCR, gel purified and sequenced. Superposition of peaks in the RIG-I KO clone sequence indicates that different alleles bear different mutations.

(E, H, K) Sequences generated in (D), (G) and (J) were analyzed with TIDE software. This algorithm decomposes the sequencing data to determine the spectrum of indels and their frequencies. The graphs show the number of nucleotides inserted or deleted and percentage of sequences affected. One nucleotide was inserted into one allele of the *RIG-I* gene, inducing a frameshift and therefore a modification of the amino acid sequence from position 51, potentially giving rise to a protein containing 86 aa. In the second allele, 10 nucleotides were deleted, also inducing a modification of the amino acid sequence from position 51 and

potentially giving rise to a protein containing 73 aa. The insertion of one nucleotide into the *IFHI1* gene (encoding MDA5) creates a frameshift at position 8, potentially giving rise to a protein containing 58 aa. In the case of MAVS, the deletion of one nucleotide induced a frameshift and therefore a different amino acid sequence from position 172, potentially giving rise to a protein containing 189 aa.

(F,L) p125-HEK cells of the indicated genotype were treated with 140 U/ml of IFNα overnight, then lysed in a buffer containing 1% of NP-40. Protein expression was tested by Western blot using the indicated antibodies.

(I) MDA5 KO and WT p125-HEK cells were infected overnight with Sendai virus at an MOI of 0.5, then lysed in a buffer containing 0.5% of NP40. MDA5 and actin expression were analyzed by Western blot.

(M) MAVS KO and WT p125-HEK cells were treated with 200 U/ml of IFNα overnight, before transfection with 100ng per well of pcDNA3 plasmid expressing human MAVS or an empty pcDNA3 vector as control. F-Luc activity in cell lysates was determined 24 hours after transfection. Data are shown as fold change compared to cells treated with transfection reagent only.

(N) RIG-I KO, MDA5 KO, and parental p125-HEK cells were transfected with 25 ng of IVT RNA or Vero EMCV RNA (CIP). F-Luc activity in cell lysates was determined 24 hours after transfection. Data are shown as luciferase values relative to parental p125-HEK.

(O) Schematic representation of RIG-I, MDA5 and MAVS proteins. The star indicates the position targeted by the sgRNA.

Panels (B) - (M) are representative of two or more independent experiments. Panels (C) and (M) show average and SD (n=3). Panel (N) shows pooled data from two independent experiments (average and SEM (n=4)).

Supplementary Figure 2

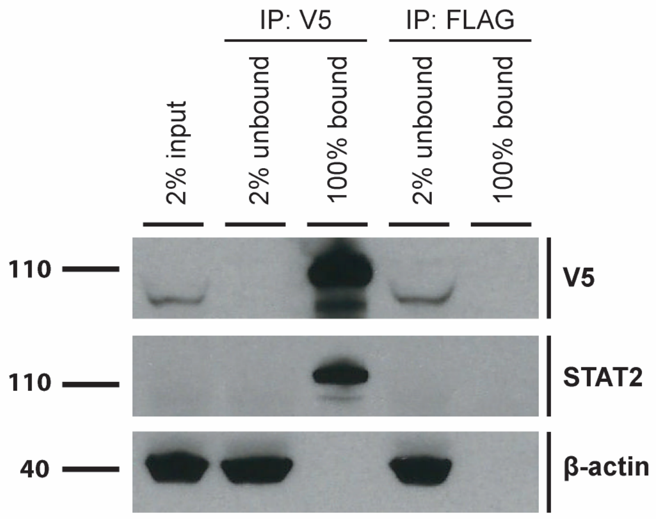

Fig. S2. ZIKV NS5 binds STAT2.

HEK293T cells were transfected with a plasmid expressing ZIKV NS5-V5. 24 hours later, cells were lysed and NS5 was immunoprecipitated with α-V5 antibody. As a negative control, α-FLAG antibody was used. Input, unbound and bound fractions were analyzed by Western blot using the indicated antibodies. Data are representative of three independent experiments.

Supplemental Table 1. Oligonucleotides.

Name	Purpose	Sequence
751_2K-NS4B_f	Cloning of 2K-NS4B	AGGAGGACAGCTATGTCTCCCCAGGACAACCAAATGGCAATCATCATCATGGTAGCAGTAGGTCTTCTGGGCTTGATTACCGCCAATGAACTCGGATGGTTGGAG
734_2K-NS4B_r	Cloning of 2K-NS4B	ACGTCTCTTGACCAAGCCAG
749_NS4A_f	Cloning of NS4A	AGGAGGACAGCTATGGGAGCGGCTTTTGGAGTG
750_NS4A_r	Cloning of NS4A	TCTTTGCTTTTCTGGCTCAGG
753_NS4A-2K-NS4B_f	Cloning of NS4A-2K-NS4B (overlap PCR)	GGCGGTAATCAAGCCCAGAAG
754_NS4A-2K-NS4B_f2	Cloning of NS4A-2K-NS4B (overlap PCR)	GGCAATCATCATCATGGTAGCAGTAGGTCTTCTGGGCTTGATTACCGCCAATGAACTCGGATGGTTGGAGAG
779_NS2B-NS3_r1	Cloning of NS2B-NS3 (overlap PCR)	CAGGCACATCCCATAGAGCACCACTCCTTTTTCCAGTCTTCACGTATACGTA
780_NS2B-NS3_f2	Cloning of NS2B-NS3 (overlap PCR)	CGTGGTACGTATACGTGAAGACTGGAAAAAGGAGTGGTGCTCTATGGGATGTGCCTG
781_NS2B-NS3_r2	Cloning of NS2B-NS3 (overlap PCR)	TCTTTTCCCAGCGGCAAACTCCTTG
797_NS2B-NS3_f1	Cloning of NS2B-NS3 (overlap PCR)	AGGAGGACAGCTATGAGCTGGCC
783_NS5-eYFP_r1	Cloning of NS5-eYFP (overlap PCR)	GCGGCCGCCACTGTGCTGGATATCAACCACTTTGTACAAGAAAGCTGGGTCGAATTCGCCCTCAGCACTCCAGGTGTAGACCCTTCTTC
784_NS5-eYFP_f2	Cloning of NS5-eYFP (overlap PCR)	TTCTTGTACAAAGTGGTTGATATCCAGCACAGTGGCGGCCGCTCGAGTCTAGAGGGGCCGCGGTTCGAAGTGAGCAAGGGCGAGGAGCTG
785_NS5-eYFP_r2	Cloning of NS5-eYFP (overlap PCR)	CAGCTCGTCCATGCCGAGAGTG
786_NS5-eYFP_f1	Cloning of NS5-eYFP (overlap PCR)	AGGAGGACAGCTATGGGGGGTG
aNLS mutant – Cluster 1 (KHK to AA) Fwd	NS5 NLS mutagenesis PCR	cctggttgtggaaagagctaggcgcagccgcacggccacgagtctgtaccaaag
aNLS mutant – Cluster 1 (KHK to AA) Rev	NS5 NLS mutagenesis PCR	ctttggtacagactcgtggccgtgcggctgcgcctagctctttccacaaccagg
aNLS mutant – Cluster 2 (RQ to AA) Fwd	NS5 NLS mutagenesis PCR	ccagacccccaagaaggcactgctgcggttatgagcatggtc
aNLS mutant – Cluster 2 (RQ to AA) Rev	NS5 NLS mutagenesis PCR	gaccatgctcataaccgcagcagtgccttcttgggggtctgg
2-O'methyltransferase mutant - Fwd	NS5 E218A cap1 mutagenesis PCR	ccgcaactctacacatgcgatgtactgggtctctg
2-O'methyltransferase mutant - Rev	NS5 E218A cap1 mutagenesis PCR	cagagacccagtacatcgcatgtgtagagttgcgg
NS5 M2634V F	Cloning of NS5 "African mutant"	atagctttgcaccaacatgggttcttcatgaccag
NS5 M2634V R	Cloning of NS5 "African mutant"	ctggtcatgaagaacccatgttggtgcaaagctat
NS5 M3392V F	Cloning of NS5 "African mutant"	cacctatgatcctgcgcatcatgttgactgtgttttta
NS5 M3392V R	Cloning of NS5 "African mutant"	taaaaacacagtcaacatgatgcgcaggatcataggtg
RIG-I sgRNA Fwd	Cloning of RIG-I sgRNA	CACCGgaacaacaagggcccaatgg
RIG-I sgRNA Rev	Cloning of RIG-I sgRNA	AAACccattgggcccttgttgttcC
MAVS sgRNA Fwd	Cloning of MAVS sgRNA	CACCggccaccatctggattcctt
MAVS sgRNA Rev	Cloning of MAVS sgRNA	AAACaaggaatccagatggtggcc

MDA-5 sgRNA Fwd	Cloning of MDA5 sgRNA	CACCGtagcggaaattctcgtctg
MDA-5 sgRNA Rev	Cloning of MDA5 sgRNA	AAACcagacgagaatttccgctaC
RIG-I KO gDNA sequencing Fwd	Validation of RIG-I targeting	ttacattgtctcagactaagaggc
RIG-I KO gDNA sequencing Rev	Validation of RIG-I targeting	Gtgaagaatgggcacagtcggcc
MAVS KO gDNA sequencing Fwd	Validation of MAVS targeting	Ggctgaggcctataggagatgcc
MAVS KO gDNA sequencing Rev	Validation of MAVS targeting	gcctgacccacaggatcatatgc
MDA5 KO gDNA sequencing Fwd	Validation of MDA5 targeting	cgtcattgtcaggcacagag
MDA5 KO gDNA sequencing Rev	Validation of MDA5 targeting	agctctgccactgtttttcc
IAV PR8 M F	RT-qPCR	CTTCTAACCGAGGTCGAAACGTA
IAV PR8 M R	RT-qPCR	GGTGACAGGATTGGTCTTGTCTTTA
ZIKV PE243 F	RT-qPCR	CGAGGAACATCCAGACTC
ZIKV PE243 R	RT-qPCR	ATTGGAGATCCTGAAGTTCC
