# Supplementary material for: Infection with a Brazilian isolate of Zika virus generates RIG‐I stimulatory RNA and the viral NS5 protein blocks type I IFN induction and signaling
Source: Eur J Immunol. 2018 Apr 6;48(7):1120–36. doi: 10.1002/eji.201847483 (PMC6055886; doi:10.1002/eji.201847483)
Supplement: Supplementary file 2 — Supplementary Information [file EJI-48-1120-s002.pdf]

European Journal of Immunology

Supporting Information
for
DOI 10.1002/eji.201847483

Jonny Hertzog, Antonio Gregorio Dias Junior, Rachel E. Rigby, Claire L. Donald, Alice Mayer, Erdinc Sezgin, Chaojun Song, Boquan Jin, Philip Hublitz, Christian Eggeling, Alain Kohl and Jan Rehwinkel

Infection with a Brazilian isolate of Zika virus generates RIG-I stimulatory RNA and the viral NS5 protein blocks type I IFN induction and signaling

© 2018 The Authors. *European Journal of Immunology* published by WILEY-VCH Verlag GmbH & Co. KGaA, Weinheim.

European Journal of Immunology

Infection with a Brazilian isolate of Zika virus generates RIG-I stimulatory RNA and the viral NS5 protein blocks type I IFN induction and signalling

Jonny Hertzog, Antonio Gregorio Dias Junior, Rachel E. Rigby, Claire L. Donald, Alice Mayer, Erdinc Sezgin, Chaojun Song, Boquan Jin, Philip Hublitz, Christian Eggeling, Alain Kohl and Jan Rehwinkel.

Correspondence: Dr. Jan Rehwinkel, Medical Research Council Human Immunology Unit, Medical Research Council Weatherall Institute of Molecular Medicine, Radcliffe Department of Medicine, University of Oxford, Oxford OX3 9DS, UK

Review Timeline:	Submission date:	05-Jan-2018
	First Editorial decision:	29-Jan-2018
	Revision received:	04-Mar-2018
	Accepted:	12-Mar-2018

Handling Executive Committee member: Prof. Annette Oxenius

Please note that the correspondence below does not include the standard editorial instructions regarding preparation and submission of revised manuscripts, only the scientific revisions requested and addressed.

First Editorial Decision
29-Jan-2018

Dear Dr. Rehwinkel,

Manuscript ID eji.201847483 entitled "Infection with a Brazilian isolate of Zika virus generates RIG-I stimulatory RNA and the viral NS5 protein blocks type I interferon induction and signalling" which you submitted to the European Journal of Immunology has been reviewed. The comments of the referees are included at the bottom of this letter.

A revised version of your manuscript that takes into account the comments of the referees will be reconsidered for publication. Should you disagree with any of the referees' concerns, you should address this in your point-by-point response and provide solid scientific reasons for why you will not make the requested changes.

You should also pay close attention to the editorial comments included below. **In particular, please edit your figure legends to follow Journal standards as outlined in the editorial comments. For all data, please report the number of samples per experiment (or experimental replicates). For flow cytometry data please show the full gating strategy, including the percentage of cells in the region or gate or event count. In the histograms/dot plots shown please report which fluorochromes were used and the scaling in the axis (log/lin). Failure to do this will result in delays in the re-review process.**

Please note that submitting a revision of your manuscript does not guarantee eventual acceptance, and that your revision will be re-reviewed by the referees before a decision is rendered.

If the revision of the paper is expected to take more than three months, please inform the editorial office. Revisions taking longer than six months may be assessed by new referees to ensure the relevance and timeliness of the data.

Once again, thank you for submitting your manuscript to European Journal of Immunology and we look forward to receiving your revision.

Yours sincerely,
Marta Vuerich

On behalf of
Prof. Annette Oxenius

Dr. Marta Vuerich
Editorial Office
European Journal of Immunology
e-mail: ejied@wiley.com
www.eji-journal.eu

****

Reviewer: 1

Comments to the Author
In their study "Infection with a Brazilian isolate of Zika virus generates RIG-I stimulatory RNA and the viral NS5 protein blocks type I interferon induction and signalling" Hertzog et al. decipher the mechanisms how a Brazilian isolate of Zika virus (a (+)ssRNA Flavivirus) induce type I IFN and actively blocks type I IFN

activity by its viral antagonistic proteins. To identify the recognition mechanism of viral RNA, isolated RNA from infected cells was transfected into RIG-I, MDA5 or MAVS deficient cells.

RIG-I pull-down precipitated ZIKA-RNA which shows RIG-I activation, suggesting direct interaction of RIG-I with viral RNA /RIG-I ligands. Single viral proteins were cloned into expression plasmids and screened for inhibition of the IFN inducing and sensing pathways. The authors identified NS5 as inhibitor of RIG-I and MDA5 stimuli (NS4a with mild effects). NS5 reduced response to recombinant type I IFN by inhibiting phosphorylation of STAT1 and reduction of STAT2 levels.

This study is a comprehensive and straightforward approach to characterize the molecular mechanisms involved in Zika virus recognition and interaction of Zika virus antagonists with the innate immune system.

Major comments

1. The results suggest a minor role of MDA5 in Zika recognition. The autors should show that their RIG-I deficient HEK293 cells have a robust response to MDA5 by stimulation with an MDA5 ligand (poly(I:C)). Comparison with transfected poly(I:C) would help to estimate the real role of MDA5 in Zika recognition.

2. STAT2 levels are reduced. However, it was not proven that STAT2 is degraded. The authors should show degradation or rephrase their statement.

3. Fig. 7 C/D; abstract: The experiment and statement involving the E218A NS5 mutant is misleading: If the virus genome is absent there is no substrate and consequently no effect for the viral cap1 methyltransferase activity. If the authors want to state that the cap1 2'O-methyltransferase has no impact on the innate immunorecognition as suggested in results and in the abstract they need to test the whole virus or a replicon system with the NS5 mutant (Daffis et al., 2010; Züst et al., 2011; Schuberth-Wagner et al., 2015). Without that experiment the authors should remove the statement from the abstract. The current experiment without viral RNA produced by a NS5 E218A mutant does not make sense and should be removed from the manuscript.

Minor comment

The authors should cite more original literature in the discussion or more recent reviews about immune recognition of nucleic acids (general reviews about innate nucleic acid recognition from 2012/13 are too old).

Reviewer: 2

Comments to the Author

Hertzog and colleagues found that RNA extracted from human lung epithelial A459 cells infected with the Brazilian Zika virus strain PE243 induced IFN-beta responses upon electroporation of human embryonic kidney HEK293 cells in a MAVS-dependent manner. The Zika virus NS5 protein blocked type I IFN induction downstream of RIG-I-like receptors as well as type I IFN receptor signaling by STAT2 degradation and inhibition of STAT1 phosphorylation.

Although the results presented in this study are not entirely surprising, the study was carried out very carefully and the presented data are convincing. The novelty about this study is that the authors analyzed the Brazilian Zika virus strain PE243, whereas other studies focused primarily on African and French Polynesian Zika virus strains. It is interesting to see that upon expression in HEK293T cells, the NS5 protein is found in fluidic dot-like structures within the nucleus. The manuscript is very well written and the relevant literature is cited in the discussion.

Minor point:

In Fig. 1B the light grey bar is marked only with “MAVS” instead of “MAVS KO”.

First Revision – authors' response

04-Mar-2018

Point-by-point response

eji.201847483

Infection with a Brazilian isolate of Zika virus generates RIG-I stimulatory RNA and the viral NS5 protein blocks type I interferon signalling

corresponding author: Jan Rehwinkel

The authors wish to thank both reviewers for critically evaluating our study and for their helpful comments and suggestions. We have included additional data on the response of our HEK293 RIG-I-KO cells to an MDA5 stimulus and rephrased parts of the manuscript as suggested by the reviewers. **We believe these changes address the concerns of the referees**. We hope that our study is sufficiently improved for the reviewers to recommend publication in *The European Journal of Immunology*.

European Journal of Immunology

Response to referee #1:

In their study "Infection with a Brazilian isolate of Zika virus generates RIG-I stimulatory RNA and the viral NS5 protein blocks type I interferon induction and signalling" Hertzog et al. decipher the mechanisms how a Brazilian isolate of Zika virus (a (+)ssRNA Flavivirus) induce type I IFN and actively blocks type I IFN activity by its viral antagonistic proteins. To identify the recognition mechanism of viral RNA, isolated RNA from infected cells was transfected into RIG-I, MDA5 or MAVS deficient cells.
RIG-I pull-down precipitated ZIKA-RNA which shows RIG-I activation, suggesting direct interaction of RIG-I with viral RNA /RIG-I ligands. Single viral proteins were cloned into expression plasmids and screened for inhibition of the IFN inducing and sensing pathways. The authors identified NS5 as inhibitor of RIG-I and MDA5 stimuli (NS4a with mild effects). NS5 reduced response to recombinant type I IFN by inhibiting phosphorylation of STAT1 and reduction of STAT2 levels.
This study is a comprehensive and straightforward approach to characterize the molecular mechanisms involved in Zika virus recognition and interaction of Zika virus antagonists with the innate immune system.

We thank the reviewer for his / her positive and constructive assessment of our manuscript.

Major comments

1. The results suggest a minor role of MDA5 in Zika recognition. The autors should show that their RIG-I deficient HEK293 cells have a robust response to MDA5 by stimulation with an MDA5 ligand (poly(I:C)). Comparison with transfected poly(I:C) would help to estimate the real role of MDA5 in Zika recognition.

We appreciate the reviewer’s concern about the responsiveness of our HEK293 RIG-I-KO reporter cells to MDA5 stimuli. We have restructured our Supplemental Figure 1 that describes creation and validation of the HEK293 *IFN*[redacted]uciferase reporter cells and the respective knock-outs. The figure now includes panel (N) that shows the response of the parental, RIG-I-KO and MDA5-KO cells to RIG-I- and MDA5-stimulatory nucleic acids. RIG-I-KO cells failed to respond to the RIG-I agonist IVT-RNA while parental and MDA5-KO cells responded to similar extent. As per the reviewer’s request, transfection of the MDA5 stimulus Vero-EMCV-RNA (CIP) resulted in a robust response in parental and RIG-I-KO cells but not in MDA5-KO cells. We used Vero-EMCV-RNA (CIP) as a selective MDA5 stimulus, given that poly(I:C) activates both RIG-I and MDA5 (Goubau et al, Nature 2014 and Kato et al, JEM 2008). We included information on the generation of the MDA5 agonist in the methods section and figure legend. Furthermore, we removed statements on the relative importance of RIG-I and MDA5. We believe these revisions address the reviewer’s concerns.

2. STAT2 levels are reduced. However, it was not proven that STAT2 is degraded. The authors should show degradation or rephrase their statement.

We agree with the reviewer and have rephrased the manuscript accordingly.

3. Fig. 7 C/D; abstract: The experiment and statement involving the E218A NS5 mutant is misleading: If the virus genome is absent there is no substrate and consequently no effect for the viral cap1 methyltransferase activity. If the authors want to state that the cap1 2'O-methyltransferase has no impact on the innate immunorecognition as suggested in results and in the abstract they need to test the whole virus or a replicon system with the NS5 mutant (Daffis et al., 2010; Züst et al., 2011; Schuberth-Wagner et al., 2015). Without that experiment the authors should remove the statement from the abstract. The current experiment without viral RNA produced by a NS5 E218A mutant does not make sense and should be removed from the manuscript.

We apologise that the description of this experiment was misleading. We did not attempt to make any conclusion on whether the capping of viral RNAs by NS5 explains the block of IFNAR signalling. It is

possible that NS5 methylates some cellular RNAs in addition to its canonical viral RNA substrate. This, at least theoretically, may relate to its function blocking IFNAR signalling. Therefore, our intention with these experiments was to test if, in an overexpression setting, NS5 requires a functional 2'O-methyltransferase domain to block IFNAR signalling. This was not the case and we believe this result should be kept in the manuscript. We have, however, as per the reviewer's request, removed the corresponding statements from the abstract and introduction, such that the point is not overstated, and clarified the text in the results section.

Minor comment
1. The authors should cite more original literature in the discussion or more recent reviews about immune recognition of nucleic acids (general reviews about innate nucleic acid recognition from 2012/13 are too old).

We replaced references 13 and 14 (reviews from 2012/3) with more recent reviews by leading experts in the field (G. Hartmann and K. Fitzgerald).

Response to referee #2:

Hertzog and colleagues found that RNA extracted from human lung epithelial A459 cells infected with the Brazilian Zika virus strain PE243 induced IFN-beta responses upon electroporation of human embryonic kidney HEK293 cells in a MAVS-dependent manner. The Zika virus NS5 protein blocked type I IFN induction downstream of RIG-I-like receptors as well as type I IFN receptor signaling by STAT2 degradation and inhibition of STAT1 phosphorylation.
Although the results presented in this study are not entirely surprising, the study was carried out very carefully and the presented data are convincing. The novelty about this study is that the authors analyzed the Brazilian Zika virus strain PE243, whereas other studies focused primarily on African and French Polynesian Zika virus strains. It is interesting to see that upon expression in HEK293T cells, the NS5 protein is found in fluidic dot-like structures within the nucleus. The manuscript is very well written and the relevant literature is cited in the discussion.

We thank the reviewer for taking the time to assess our manuscript and her/his positive comments.

Minor point:
1. In Fig. 1B the light grey bar is marked only with "MAVS" instead of "MAVS KO".

We thank the reviewer for recognising this omission and have corrected it.

Second Editorial Decision

06-Mar-2018

Dear Dr. Rehwinkel,

It is a pleasure to provisionally accept your manuscript entitled "Infection with a Brazilian isolate of Zika virus generates RIG-I stimulatory RNA and the viral NS5 protein blocks type I interferon induction and signalling" for publication in the European Journal of Immunology. For final acceptance, please follow the instructions below and return the requested items as soon as possible as we cannot process your manuscript further until all items listed below are dealt with.

European Journal of Immunology

Please note that EJI articles are now published online a few days after final acceptance (see Accepted Articles: http://onlinelibrary.wiley.com/journal/10.1002/(ISSN)1521-4141/accepted). The files used for the Accepted Articles are the final files and information supplied by you in Manuscript Central. You should therefore check that all the information (including author names) is correct as changes will NOT be permitted until the proofs stage.

We look forward to hearing from you and thank you for submitting your manuscript to the European Journal of Immunology.

Yours sincerely,
Eloho Etemire

on behalf of
Prof. Annette Oxenius
Dr. Eloho Etemire
Editorial Office
European Journal of Immunology
e-mail: ejied@wiley.com
www.eji-journal.eu
